# Mechanism of promoting the regeneration of oral tissues by injectable hydrogel

**DOI:** 10.3205/dgkh000588

**Published:** 2025-09-30

**Authors:** Karthik Shunmugavelu, Bala Geetha Shakthi Chakravarthy, Shaila Umachandran

**Affiliations:** 1 Department of Dentistry, PSP Medical College Hospital and Research Institute, Tambaram, Tamilnadu, India; 2 Department of community medicine SRM Medical College Hospital & Research centre, SRM Institute of Science & Technology, Tamil Nadu, India; 3 Department of Community Medicine, Sree Balaji Medical College and Hospital Chrompet, Chennai, India

**Keywords:** injectable hydrogel, oral tissue regeneration, tissue engineering, periodontal repair, biomaterials, regenerative medicine.

## Abstract

**Introduction:**

The regeneration of oral tissues, including periodontal ligaments, alveolar bone, and soft tissues, remains a major challenge in dentistry and maxillofacial surgery. Traditional approaches, such as grafting and guided tissue regeneration, are limited by donor site morbidity, immune responses, and surgical complexities. Injectable hydrogels have emerged as promising biomaterials due to their ability to deliver cells, growth factors, and bioactive molecules directly to injury sites in a minimally invasive manner. Their adjustable properties and ability to mimic the extracellular matrix make them ideal for promoting tissue repair and regeneration. This review evaluates the literature on injectable hydrogels in oral tissue regeneration, with a focus on their composition, mechanism of action, and clinical applications.

**Methods:**

A systematic search was conducted across PubMed, Scopus, Web of Science, and Google Scholar for studies published between 2000 and 2024, following PRISMA guidelines.

**Results:**

Thirty (30) studies met the inclusion criteria, with five selected for detailed analysis. The findings highlight the regenerative potential of hydrogels composed of natural polymers, e.g., collagen, alginate, and hyaluronic acid, synthetic polymers, e.g., polyethylene glycol PEG, and polycaprolactone, as well as poly(lactic-co-glycolic)acid. Advanced hydrogel formulations, including self-healing, thermosensitive, and bioactive hydrogels, demonstrate enhanced biocompatibility, mechanical properties, and controlled drug delivery.

**Conclusion:**

Despite their potential, challenges such as long-term stability, clinical translation, and standardization in hydrogel formulations remain. Further research is required to optimize hydrogel-based therapies for widespread clinical use in oral and periodontal tissue regeneration.

## Zusammenfassung

**Hintergrund:**

Die Regeneration von oralem Gewebe einschließlich parodontaler Ligamente, Alveolarknochen und Weichgewebe, ist nach wie vor eine große Herausforderung in der Mund- Kiefer-Gesichtschirurgie. Herkömmliche Ansätze wie Transplantation und gesteuerte Geweberegeneration werden durch Morbidität an der Spenderstelle, Immunreaktionen und chirurgische Komplexität eingeschränkt. Injizierbare Hydrogele haben sich als vielversprechende Biomaterialien erwiesen, da sie in der Lage sind, Zellen, Wachstumsfaktoren und bioaktive Moleküle auf minimalinvasive Weise direkt an verletzte Stellen zu bringen. Aufgrund ihrer einstellbaren Eigenschaften und ihrer Fähigkeit, die extrazelluläre Matrix zu imitieren, sind sie ideal für die Förderung der Gewebereparatur und -regeneration. In dieser Übersicht wird die Literatur zu injizierbaren Hydrogelen für die orale Geweberegeneration ausgewertet, wobei der Schwerpunkt auf ihrer Zusammensetzung, ihrem Wirkmechanismus und ihren klinischen Anwendungen liegt.

**Methode:**

Es wurde eine systematische Suche in PubMed, Scopus, Web of Science und Google Scholar nach Studien durchgeführt, die zwischen 2000 und 2024 veröffentlicht wurden, wobei die PRISMA-Richtlinien eingehalten wurden.

**Ergebnisse:**

30 Studien erfüllten die Einschlusskriterien, von denen fünf für eine detaillierte Analyse ausgewählt wurden. Die Ergebnisse unterstreichen das regenerative Potenzial von Hydrogelen, die aus natürlichen Polymeren, z.B. Kollagen, Alginat und Hyaluronsäure, synthetischen Polymeren, z.B. Polyethylenglycol und Polycaprolacton, sowie Poly(lactic-co-glycolid) bestehen. Fortschrittliche Hydrogelformulierungen, einschließlich selbstheilender, thermosensitiver und bioaktiver Hydrogele, weisen eine verbesserte Biokompatibilität, mechanische Eigenschaften und eine kontrollierte Wirkstoffabgabe auf.

**Schlussfolgerung:**

Trotz ihres Potenzials bleiben Herausforderungen wie Langzeitstabilität, klinische Umsetzung und Standardisierung von Hydrogelformulierungen bestehen. Weitere Forschungsarbeiten sind erforderlich, um Hydrogel-basierte Therapien für den breiten klinischen Einsatz bei der Regeneration von oralem und parodontalem Gewebe zu optimieren.

## Introduction

Regeneration of oral tissues, including periodontal ligaments, alveolar bone, and soft tissues, remains a significant challenge in dentistry and maxillofacial surgery. Traditional regenerative approaches, such as grafts and guided tissue regeneration, present limitations in terms of donor site morbidity and immune responses. Injectable hydrogels have emerged as promising biomaterials due to their capacity to deliver cells, growth factors, and bioactive molecules directly to the injury site. Their minimally invasive application, tunable properties, and ability to mimic the extracellular matrix (ECM) make them ideal for facilitating tissue repair and regeneration. This review aims to systematically evaluate the available literature on injectable hydrogels in oral tissue regeneration, focusing on their composition, mechanism of action, and clinical implications.

## Methods

A systematic search was conducted in PubMed, Scopus, Web of Science, and Google Scholar for relevant studies published between 2000 and 2024. The preferred reporting items for systematic reviews and meta-analyses (PRISMA) guidelines were followed. The inclusion criteria were:

- Studies evaluating the role of injectable hydrogels in oral tissue regeneration,
- In-vivo and in-vitro studies investigating the biological mechanisms and outcomes,
- Clinical studies assessing the efficacy of hydrogel-based treatments.

Exclusion criteria included:

- Studies focusing on non-injectable hydrogels,
- Reviews, meta-analyses, and non-English language articles.

Data were extracted on hydrogel composition, bioactive agents, mode of application, tissue response, and clinical outcomes. A risk-of-bias assessment was performed using the Cochrane Risk of Bias tool.

## Results

The literature search yielded 85 studies, out of which 30 met the inclusion criteria. Five (5) among them were used for the detailed analysis. Injectable hydrogels composed of natural (e.g., collagen, alginate, hyaluronic acid) and synthetic polymers (e.g., polyethylene glycol [PEG], polycaprolactone [PCL], and poly(lactic-co-glycolic acid) [PLGA]) demonstrated significant regenerative potential (Table 1 (Tab. 1)). 

## Discussion

Tang et al. [1] provided a comprehensive review of chitosan-based injectable hydrogels, emphasizing their potential in bone and dental tissue engineering. The study highlighted the limitations of conventional bone repair approaches, e.g., autografts and allografts, which are associated with complications such as donor site morbidity, immune rejection, and disease transmission. In contrast, bone tissue engineering (BTE) is presented as a safer and more effective alternative, with chitosan standing out due to its low immunogenicity, biodegradability, and cost-effectiveness. The review elaborated on the advantages of chitosan-based injectable hydrogels, including their thermo/pH responsiveness, high water absorption capacity, and minimally invasive application. Additionally, chitosan was found to form porous networks that facilitate tissue integration and mold into irregular defects. The study underscored the significance of composite formulations incorporating natural or synthetic polymers and bioactive agents to enhance the efficacy of chitosan-based hydrogels. The findings provided valuable insights into the physicochemical properties, preparation methods, and future research directions for developing next-generation scaffold materials for improved dental and orthopedic applications.

Mehrotra et al. [2] explored the transition from injectable hydrogels to 3D-printed hydrogel-based scaffolds in maxillofacial tissue engineering. The study emphasized the superior mechanical strength, biocompatibility, and biochemical interactions of hydrogel-based scaffolds, which are essential for effective tissue regeneration. Injectable hydrogels were highlighted for their ability to facilitate minimally invasive procedures, whereas 3D-printed hydrogels allowed for precise structural design and controlled drug delivery. The review also discussed advancements in self-healing and shape-memory hydrogels, which significantly enhance functional outcomes in bone defect repair, periodontal regeneration, and cartilage reconstruction. The findings indicated that the combination of injectability and 3D printing could lead to more patient-specific and adaptable solutions in maxillofacial applications, making them promising alternatives for clinical translation.

Haugen et al. [3] examined the role of injectable biomaterials in dental tissue regeneration, emphasizing their advantages over pre-formed scaffolds. The study highlighted the importance of these biomaterials in addressing small, confined, and hard-to-reach defects in the maxilla-oral region, where traditional methods often prove inadequate. A range of biomaterials was analyzed for their biocompatibility, integration capacity, and healing potential. Notably, the study discussed the contribution of nanofibers in dental tissue engineering, as they enhance structural support, bioactivity, and the controlled release of growth factors. Injectable biomaterials, when integrated with tissue engineering approaches, were shown to restore dental tissue functions such as periodontal ligament and pulp tissue regeneration. The study underscored the potential of these biomaterials for future clinical applications and called for further research into biodegradable, bioactive, and smart injectable scaffolds for enhanced mechanical properties and long-term success.

Bertsch et al. [4] reviewed the advancements in self-healing injectable hydrogels, a novel class of biomaterials that enhance the durability and adaptability of tissue engineering applications. These hydrogels, developed based on reversible chemistry, allow for temporary fluidization under shear stress and recovery of mechanical properties post-injection, making them particularly beneficial for tissue regeneration. The study emphasized their advantages, including minimally invasive application via syringe, moldability for patient-specific interventions, and enhanced tissue integration. Additionally, self-healing hydrogels were found to provide mechanical support, facilitate controlled therapeutic delivery, and recruit host cells to improve natural healing. The research highlighted their applications in advanced tissue engineering and regenerative medicine, including 3D printing of complex tissues and organoids. The findings suggest that self-healing hydrogels could revolutionize biomedical applications by offering long-term stability and improved functional outcomes.

El-Nablaway et al. [5] addressed the challenges in treating periodontitis, a complex inflammation-related disease that involves an interplay between an infectious microbiome and host immune responses. The study highlighted the limitations of conventional treatments, particularly the difficulty in sustaining therapeutic drug levels due to natural oral processes like saliva production and mastication. The research focused on the development of bioactive injectable mucoadhesive thermosensitive hydrogels, which offer biocompatibility, biodegradability, and prolonged drug delivery for periodontal tissues. The emergence of intelligent thermosensitive hydrogels, capable of undergoing sol-gel transitions in response to local temperature changes, was highlighted as a breakthrough in targeted drug delivery. The study emphasized the potential of smart hydrogel-based treatment approaches in enhancing therapeutic efficacy while minimizing systemic side effects. Future research directions included the development of more effective hydrogel systems for periodontal therapy and clinical validation for widespread use in dentistry.

The reviewed studies collectively highlight the significant advancements in injectable hydrogels for bone and dental tissue regeneration. While chitosan-based and self-healing hydrogels have demonstrated promising biocompatibility and bioactivity, 3D-printed hydrogels have enabled precise tissue engineering with controlled drug release. Furthermore, the integration of nanofibers and thermosensitive properties has enhanced hydrogel adaptability for specific dental applications. However, challenges remain in terms of optimizing mechanical properties, ensuring long-term stability, and translating these innovations into clinical practice. Future research should focus on the development of multifunctional biomaterials that integrate self-healing, smart drug delivery, and patient-specific customization to advance the field of regenerative medicine and tissue engineering.

## Conclusion

The advancements in injectable hydrogels for bone and dental tissue engineering underscore their transformative potential in regenerative medicine. Chitosan-based hydrogels, self-healing formulations, and 3D-printed hydrogel scaffolds have emerged as promising alternatives to traditional bone repair methods, offering enhanced biocompatibility, biodegradability, and structural adaptability. The integration of nanofibers, bioactive agents, and thermosensitive properties further improves their functional applications, particularly in maxillofacial and periodontal tissue regeneration. Despite these significant strides, challenges persist in optimizing mechanical properties, achieving sustained therapeutic effects, and ensuring clinical translation. Future research should prioritize the development of multifunctional biomaterials that incorporate smart drug delivery, patient-specific customization, and long-term stability to bridge the gap between experimental success and widespread clinical adoption. By addressing these challenges, injectable hydrogel technology has the potential to revolutionize tissue engineering and improve patient outcomes in dental and orthopedic applications.

## Notes

### Author’s ORCID

- Shunmugavelu K: https://orcid.org/0000-0001-7562-8802
- Shakthi Chakravarthy BG: https://orcid.org/0009-0006-0582-1563

### Funding

None.

### Competing interests

The authors declare that they have no competing interests.

## Figures and Tables

**Table 1:**
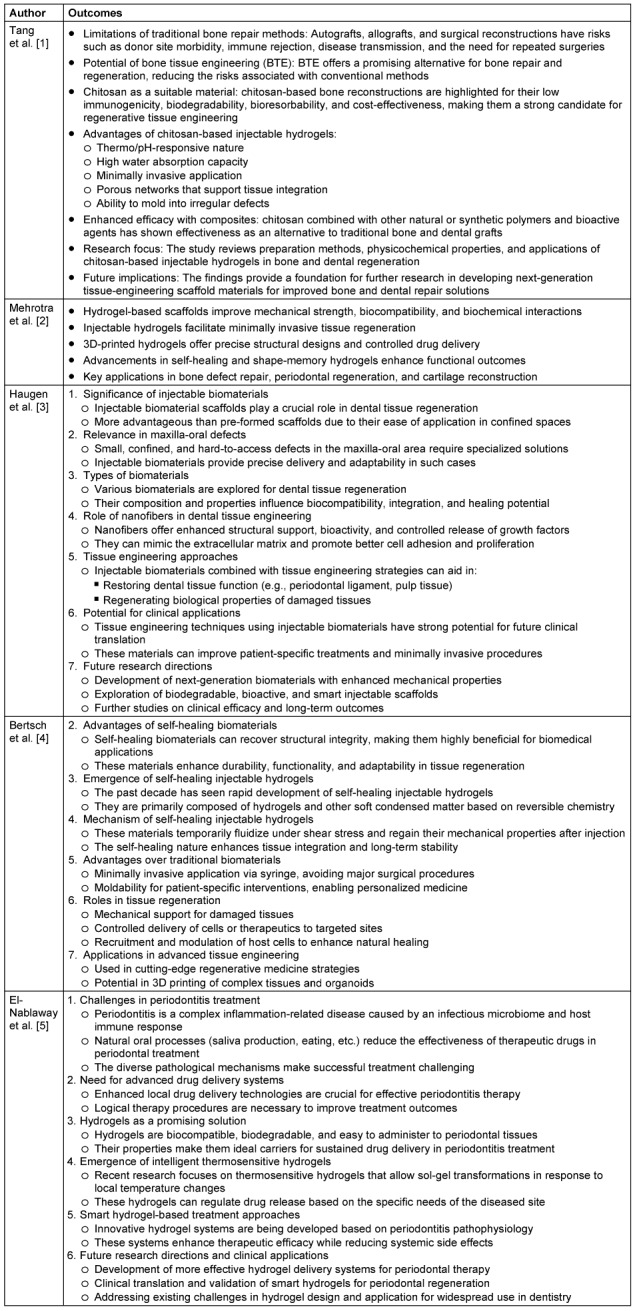
An overview of application of injectable hydrogel in regeneration of oral tissues
